# Salvage Splenopexy for Torsion of Wandering Spleen in a Child

**Published:** 2014-04-01

**Authors:** Ram Babu Goyal, Rahul Gupta, Girish Prabhakar, Praveen Mathur, Tariq Ahmed Mala

**Affiliations:** Department of Paediatric Surgery, SPMCHI, SMS Hospital, Jaipur India

**Keywords:** Splenic torsion, Wandering spleen, Salvage splenopexy

## Abstract

The wandering spleen is a rare condition characterized by the absence or underdevelopment of one or all of the splenic suspensory ligaments that resulting in increased splenic mobility and rarely torsion. Preventing infarction is the aim of a prompt surgery by splenopexy. We report a case of salvage splenopexy in torsion of a wandering spleen in a three year old girl presented with severe abdominal pain for three days.

## INTRODUCTION

Wandering spleen was first described by Van Horne, a Dutch physician, in 1667 on autopsy. In 1877, Martin, a German obstetrician, performed the first splenectomy for a wandering spleen. In 1895, LudwikRydygier performed the first splenopexy to immobilize a wandering spleen, by fixing it to the peritoneum.[1]Approximately 500 cases have been reported in the literature; less than one-third reported in children.[1,2]Wandering spleen lacks lienorenal and/or gastrosplenic ligaments; instead, it has long vascular pedicle that can twist creating parenchymal congestion that may progress to infarction if not properly addressed. Herein, we describe a case of wandering spleen in a three year old girl which was addressed with salvage splenopexy.

## CASE REPORT

A 3-year-old girl presented with severe abdominal pain for 3 days. There was no history of trauma. There was a history of chronic vague lower abdominal pain for which she had multiple visits to the general practitioners. On examination, she was afebrile, pulse rate120/min, and dehydrated. Abdomen was tense with tenderness. A mobile mass in the umbilical region was palpable. A digital rectal examination revealed finger staining of blood with mucus. Complete blood count was normal and abdominal radiographs were inconclusive. Ultrasonography (USG) of abdomen showed hypoechoic mass in the umbilical region. The cause of acute abdomen could not be established preoperatively. On exploration; an enlarged, dark, and congested spleen was present in the mid abdomen. The splenic pedicle (15 cm long) was twisted clockwise (Fig. 1). There was absence of all suspensory ligaments and short gastric vessels. The spleen was de-twisted. Color of spleen started changing to red in patchy areas. Omentum was wrapped over spleen and it was fixed to anterior abdominal wall in left hypochondrium (Fig. 2). Postoperative course was uneventful. USG confirmed the normal anatomical position of well fixed spleen. Splenic biopsy 4 months after the procedure was performed and it revealed viable splenic tissue with some areas of fibrosis. She is doing well on follow-up.

**Figure:**
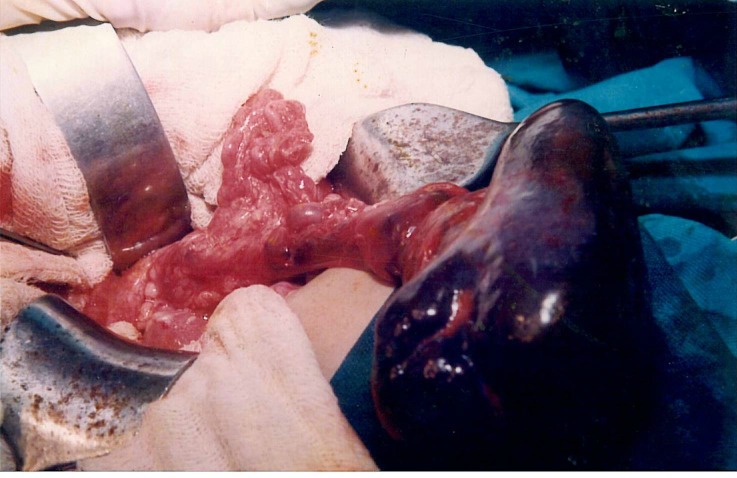
Figure 1:Operative photograph showing a large congested spleen in the umbilical region, with no ligamentous attachments and is twisted 360 degrees clockwise on its vascular pedicle.

**Figure:**
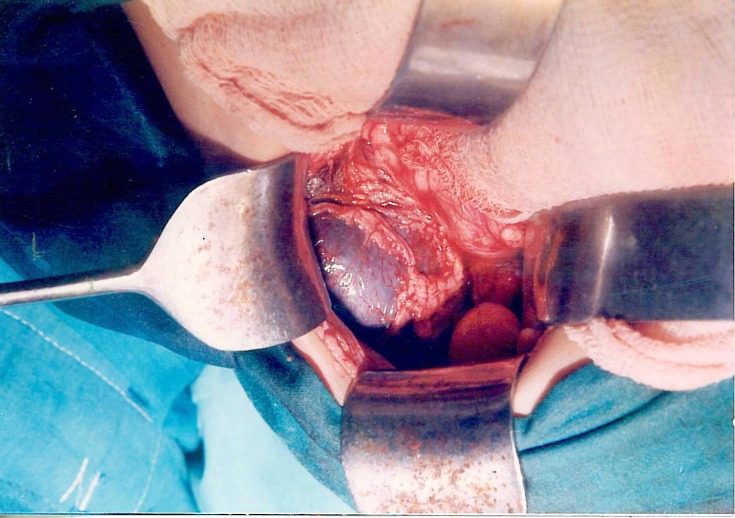
Figure 2:Operative photograph after omental wrapping and splenopexy to anterior abdominal wall.

## DISCUSSION

Wandering spleen has occasionally been seen in association with other conditions in which normal intra-abdominal fixation has not occurred (prune belly syndrome, renal agenesis, gastric volvulus, diaphragmatic eventration, and congenital diaphragmatic hernia.[2]). Acquired etiologies include laxity of ligaments normally attached to spleen (such as in multiparity, hormonal effects of pregnancy, generalized connective tissue diseases, and visceroptosis), splenomegaly (due to malaria, Hodgkin’s disease, lipid storage diseases), and abdominal trauma.[3-5]The incidence of wandering spleen remains unknown. It is most commonly found in women of reproductive age.[4]Wandering spleen may remain asymptomatic or may present clinically in the form of a freely mobile lump in abdomen.[4] Sudden torsion may result in acute abdomen with life threatening complications with a mortality rate as high as 50%.[4,5]Patients may also present with chronic pain with partial torsion followed by spontaneous detorsion [6]. In our case there was a history of vague chronic abdominal pain which might point towards partial torsion.

Earlier, the treatment for a wandering spleen with or without torsion was splenectomy [5, 6]. Currently, preservation of the spleen with splenopexy is desirable, and is highly recommended in very young patients to avoid overwhelming postsplenectomy infection (OPSI). Successful salvage splenopexy in torsion of wandering spleen has been reported in few cases only.[6-10]. Splenectomy is performed only when there is no evidence of splenic blood flow after detorsion of spleen, or when it is infarcted, in danger of rupture, and with splenic vein thrombosis [7]. Salvage splenopexy may involve fixing the spleen via its pedicle, to the anterior abdominal wall or diaphragm, creating an in-situ tissue pouch using omentum, transposition of the colonic flexure and gastrocolic ligament, suturing a basket of mesh around the spleen and auto transplantation of viable splenic tissue in greater omentum or retroperitoneum [5-9]. We performed the technique described by Maxwell-Armstrong and Stringel, by wrapping spleen with omentum and fixing it to anterior abdominal wall.[7,8].

To conclude, torsion of a wandering spleen is a rare cause of an acute abdomen in pediatric patients. Diagnosis is extremely difficult because of its rarity and nonspecific findings. Optimal treatment requires a heightened awareness and prompt surgical intervention. Splenopexy with splenic salvage is a safe procedure in torsion of a paediatric wandering spleen, as long as there is no evidence of infarction.

## Footnotes

**Source of Support:** Nil

**Conflict of Interest:** None declared

